# How did the Covid-19 Pandemic Impact the Life of Patients with Schizophrenia Spectrum Disorders?

**DOI:** 10.1192/j.eurpsy.2022.1327

**Published:** 2022-09-01

**Authors:** O. Nombora, A. Certo, J. Rodrigues, Â. Venâncio

**Affiliations:** Vila Nova de Gaia Hospital Center, Psychiatry And Mental Health Service, Vila Nova de Gaia, Portugal

**Keywords:** Covid-19 pandemic, severe mental illness, schizophrénia, Psychosis

## Abstract

**Introduction:**

Since the first outbreak, the Covid-19 pandemic has had and still has several implications worldwide, particularly in severe mentally ill patients, leading to multiple challenges in their management.

**Objectives:**

We aim to assess the impact and implications of the Covid-19 pandemic on patients with Schizophrenia Spectrum Disorders (SSD) and the treatment recommendations available.

**Methods:**

We conduct an integrative review using PubMed database. Search terms included: “psychosis” AND “COVID-19 pandemic”, “schizophrenia and COVID-19”, “severe mental illness” AND “COVID-19”. The search period was between 1^st^ January 2020 and 31^th^ July 2021.

**Results:**

Studies postulated that people with SSD are at a higher risk of COVID-19 infection with a poorer medical and social outcome which is attributed to factors such as higher rates of disadvantageous lifestyle behaviours, medical comorbidities, antipsychotic medication metabolic effects, psychosocial adversities, smaller social networks and poor engagement with general health services. The Covid-19 pandemic also demanded adjustments in treatment guidelines and monitoring, particularly in patients with SSD on Clozapine. Many studies address the importance of psychiatric care and treatment during the pandemic. They emphasize rapid implementation of measures to decrease the risk of COVID-19 transmission and maintain continuity care and research. An individualized and flexible approach is needed to promote safety of SSD patients.

**Conclusions:**

Particular attention is required by clinicians to help SSD patients face the current pandemic situation. Future epidemiological studies are needed in order to better understand the impact of the COVID-19 pandemic in this population and provide proper care.

**Disclosure:**

No significant relationships.

